# Diverse role of androgen action in human breast cancer

**DOI:** 10.1530/EO-22-0048

**Published:** 2022-08-22

**Authors:** Kiyoshi Takagi, Mio Yamaguchi, Minoru Miyashita, Hironobu Sasano, Takashi Suzuki

**Affiliations:** 1 Department of Pathology and Histotechnology, Tohoku University Graduate School of Medicine, Sendai, Japan; 2 Department of Breast and Endocrine Surgical Oncology, Tohoku University Graduate School of Medicine, Sendai, Japan; 3 Department of Anatomic Pathology, Tohoku University Graduate School of Medicine, Sendai, Japan; 4 Department of Pathology, Tohoku University Hospital, Sendai, Japan

**Keywords:** breast cancer, androgen receptor, endocrine therapy resistance, therapeutic target

## Abstract

Breast cancer is a hormone-dependent cancer, and sex steroids play a pivotal role in breast cancer progression. Estrogens are strongly associated with breast cancers, and the estrogen receptor (estrogen receptor α; ERα) is expressed in 70–80% of human breast carcinoma tissues. Although antiestrogen therapies (endocrine therapies) have significantly improved clinical outcomes in ERα-positive breast cancer patients, some patients experience recurrence after treatment. In addition, patients with breast carcinoma lacking ERα expression do not benefit from endocrine therapy. The androgen receptor (AR) is also expressed in >70% of breast carcinoma tissues. Growing evidence supports this novel therapeutic target for the treatment of triple-negative breast cancers that lack ERα, progesterone receptor, and human EGF receptor 2, and ERα-positive breast cancers, which are resistant to conventional endocrine therapy. However, the clinical significance of AR expression is still controversial and the biological function of androgens in breast cancers is unclear. In this review, we focus on the recent findings concerning androgen action in breast cancers and the contributions of androgens to improved breast cancer therapy.

## Introduction

Breast cancer is the most common malignancy in women. Breast cancer is not a single disease but a heterogeneous disease with highly complicated gene expression profiles. Sex steroids are associated with the biological features of breast cancers, and estrogens significantly contribute to breast cancer progression. Antiestrogen therapies, such as selective estrogen receptor (ER) modulators (SERMs) like tamoxifen, selective ER degraders like fulvestrant, aromatase inhibitors (AIs), and luteinizing hormone-releasing hormone agonists, are currently available and improve clinical outcomes in ERα-positive breast cancer patients. However, acquired endocrine therapy resistance leads to recurrence in up to 20% of patients with ERα-positive breast cancers (Waks & Winer 2019). Triple-negative breast cancers (TNBCs) lack ERα, progesterone receptor (PR), and human EGF receptor 2 (HER2). High-risk breast cancers like TNBCs are also treated with chemotherapy, but 25% of patients develop distant recurrence after adjuvant chemotherapy (Tevaarwerk *et al.* 2013). Therefore, new therapeutic targets for breast cancers are being explored.

In addition to ERα, the androgen receptor (AR) is also expressed in 70–90% of breast carcinomas (Takagi *et al.* 2018). Although androgens suppress the growth of breast cancers, growing evidence supports a pro-tumorigenic role for androgens in both ERα-positive and ERα-negative breast cancers. Thus, AR is a potential new therapeutic target in breast cancers. In this review, we summarize recent findings concerning the role and regulation of androgens in breast cancers.

## Intratumoral synthesis and metabolism of androgens

**Figure 1:**
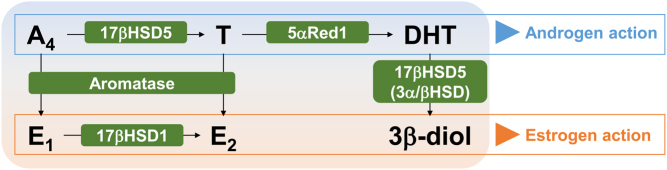
Scheme of intratumoral androgen synthesis. Dihydrotestosterone (DHT), a biologically active androgen, is locally produced by enzymes such as 17β-hydroxysteroid dehydrogenase type 5 (17βHSD5) and 5α-reductase 1 (5αRed1). Aromatase negatively regulates intratumoral DHT synthesis by converting testosterone (T) to estradiol (E_2_). DHT is metabolized to 5α-androstane-3β,17β-diol (3β-diol), which possesses estrogenic effects. A_4_, androstenedione; E_1_, estrone.

Dihydrotestosterone (DHT) has the highest affinity for AR and causes nuclear translocation of AR, leading to activation of transcriptional activity. Interestingly, tissue concentrations of DHT are higher in breast carcinoma tissues than in non-neoplastic breast tissues (Shibuya *et al.* 2008), indicating intratumoral DHT synthesis in breast carcinoma tissues. To better understand the intratumoral synthesis of androgens in human malignancies, tissue concentrations of androgens should be measured by accurate and sensitive methods, such as mass spectrometry-based assays (Shibuya *et al.* 2008, Takagi *et al.* 2010, Tanaka *et al.* 2015, Choi 2018, Hashimoto *et al.* 2018), and the enzymes responsible for androgen synthesis should be immunolocalized (Fig. 1). Of note, these enzymes are expressed regardless of ERα status and DHT may be synthesized in both ERα-positive and ERα-negative breast carcinoma tissues (Suzuki *et al.* 2001, 2005).

The circulating adrenal androgen, androstenedione, is converted to testosterone by 17β-hydroxysteroid dehydrogenase type 5 (17βHSD5), a member of the aldo-keto reductase superfamily known as AKR1C3. Testosterone is then reduced to the biologically active androgen, DHT, by 5α-reductase (5αRed). In addition to testosterone synthesis, 17βHSD5 also has 3αHSD and 3βHSD activities (Steckelbroeck *et al.* 2004) that contribute to DHT metabolism to 5α-androstane-3β,17β-diol (3β-diol). Of note, 3β-diol acts as an estrogen rather than an androgen, and activation of ERα by 3β-diol is closely associated with resistance to endocrine therapy (Hanamura *et al.* 2013, 2016, described in more detail later). In addition, 17βHSD5 acts as a prostaglandin (PG) F2α synthase and reduces PGD2 to 11β-PGF2α (Yoda *et al.* 2015), which promotes proliferation and epithelial–mesenchymal transition (EMT) of breast cancers (Yoda *et al.* 2015). However, conflicting data concerning expression levels of 17βHSD5 in breast carcinoma tissues have been reported; thus, the role of 17βHSD5 is unclear. Recent public database analysis revealed that 17βHSD5 (AKR1C3) mRNA expression is decreased in breast cancer tissue compared to normal breast tissues (Yin *et al.* 2014) but is significantly upregulated in tamoxifen-resistant breast cancers (Xu *et al.* 2021).

The expression of 5αRed, which reduces testosterone to DHT, is elevated in breast carcinoma tissues compared to non-neoplastic breast tissues (Li *et al.* 2020). To date, three isoforms of 5αRed have been identified (5αRed1–3). Immunoreactivity toward 5αRed1 is frequently (58%) observed in breast carcinoma tissues, and 5αRed1 mRNA levels are eight-fold higher in breast carcinoma tissues compared with expression in non-neoplastic breast tissues. Immunoreactivity toward 5αRed2 is less frequent (15%) in breast carcinoma tissues compared with 5αRed1 (Suzuki *et al.* 2001), although 5αRed2 mRNA levels are higher in breast carcinoma tissues compared with expression levels in non-neoplastic breast tissues (Suzuki *et al.* 2001). In addition, we demonstrated that intratumoral DHT concentrations significantly correlate with 5αRed1 but not 5αRed2 immunoreactivity (Suzuki *et al.* 2007). Thus, 5αRed1 is mainly responsible for intratumoral DHT synthesis in breast cancers. In 2008, 5αRed3 was identified in prostate carcinoma tissues (Uemura *et al.* 2008). The contribution of 5αRed3 to DHT synthesis and the clinical significance of 5αRed3 in breast cancer are largely unclear. However, a recent database analysis showed that overexpression of 5αRed3 correlates with lymph node metastasis and shorter overall survival of breast cancer patients (Zhang *et al.* 2021). In contrast, 5αRed1 negatively correlates with histological grade and tumor size in breast carcinomas (Suzuki *et al.* 2001).

Aromatase converts testosterone to estradiol (E_2_). Thus, aromatase negatively regulates intratumoral DHT synthesis in breast cancer tissues. DHT concentrations are higher in ductal carcinoma *in situ* (DCIS) than in invasive ductal carcinoma tissues, where aromatase expression is higher compared to DCIS tissues (Shibuya *et al.* 2008). The AI, 4-hydroxyandrostenedione, increases intratumoral testosterone concentrations in a mammary breast cancer model (Spinola *et al.* 1988). The AI, letrozole, increases intraovarian testosterone concentrations in women and is widely used for endocrine therapy in breast cancers (Garcia-Velasco *et al.* 2005). Furthermore, we have previously reported that intratumoral DHT concentrations are significantly higher in breast cancer tissues after neoadjuvant AI exemestane therapy compared with the levels in tissues without exemestane therapy (Takagi *et al.* 2010). Exemestane also increases DHT production in human prostate microsomes measured with gas chromatography–mass spectrometry (Amaral *et al.* 2020). Therefore, intratumoral DHT synthesis and the expression of androgen-responsive genes are upregulated following AI treatment (Takagi *et al.* 2010, 2018).

## AR–ERα crosstalk in ERα-positive breast cancer

Activation of steroid receptors is initiated by ligand binding to the C-terminal ligand binding domain, which triggers steroid receptor translocation to the nucleus. Nuclear steroid receptors act as transcriptional factors by binding to enhancer regions, including the estrogen-responsive element (ERE) and androgen-responsive element. Thus, the actions of sex steroids are achieved through gene regulation. ERα and AR are co-expressed and directly interact with each other in breast cancer cells (D’Amato *et al.* 2016). Thus, crosstalk between ERα and AR and the resulting alterations in gene expression are hot topics for investigation. Recent studies indicate that estrogen/ERα signaling is suppressed by androgen/AR. For example, DHT significantly suppresses E_2_-induced gene signatures of cell cycle regulation in a patient-derived explant model from ERα-positive breast cancers (Hickey *et al.* 2021). A recent genome-wide analysis also revealed that ERα occupancy on EREs is decreased after AR activation (Ponnusamy *et al.* 2019) due to shared transcriptional coactivators. Steroid receptors often share coregulators and competition for cofactors occurs when cofactor levels are limited (cofactor squelching). For instance, mediator complex subunit 1 (MED1) has two LXXLL motifs and interacts with several nuclear receptors, including ERα and AR, to stimulate transcriptional activity (Weber & Garabedian 2018). AIB1/Src3 is a coactivator of ERα and is recruited to the *CCND1* gene promoter in MCF-7 breast cancer cells treated with E_2_; AIB1/Src3 recruitment is significantly suppressed by DHT (De Amicis *et al.* 2019). On the other hand, AR transactivation is also suppressed by estrogen (Rahim & O’Regan 2017), and we demonstrated that induction of androgen-responsive genes (*PSA, HSD17B2*) is suppressed by E_2_ (Suzuki *et al.* 2010, Takagi *et al.* 2010). In addition, we demonstrated that expression levels of approximately one-third of putative androgen-responsive genes are increased in response to AI treatment, which increases intratumoral DHT synthesis (Takagi *et al.* 2018). Interestingly, AR and ERα can also cooperate. For example, the expression levels of the UDP-glucuronosyltransferases, *UGT2B15* and *UGT2B17*, are regulated by tandem binding of AR and ERα at their proximal promoter, and DHT-induced *UGT2B15* and *UGT2B17* expression levels are significantly suppressed by both antiestrogen fulvestrant and antiandrogen flutamide in MCF-7 cells (Hu *et al.* 2016). Furthermore, the second-generation antiandrogen, enzalutamide, inhibits chromatin binding of both AR and ERα, suggesting that AR is necessary for maximum chromatin binding of ERα (D’Amato *et al.* 2016). E_2_ also induces chromatin binding of AR, but the binding sites are unique compared to DHT.

## Clinical significance of androgens in breast cancer

Many researchers have examined the effects of androgen on breast cancer cell proliferation, and both pro- and antiproliferative effects have been demonstrated. This discrepancy is partly due to different experimental conditions, such as different androgen structures (aromatizable or non-aromatizable) and cell lines. Most importantly, androgen action varies according to molecular subtypes, according to well-established immunohistochemical markers (luminal type: positive for ERα; HER2 type: ERα-negative and HER2-positive; TNBC: negative for ERα, PR, and HER2). ERα is affected by androgen actions in breast cancers. Androgens have antiproliferative effects on ERα-positive breast cancers and proliferative effects on ERα-negative breast cancers. In ERα-positive breast cancers, androgens suppress estrogen-dependent growth (Hickey *et al.* 2021), partially due to the suppressive effects of androgens/AR on ERα transactivation, as described earlier. Aromatase activity is also important; aromatizable androgens are likely to be converted to estrogens and promote the proliferation of ERα-positive breast cancers. Indeed, testosterone promotes MCF-7 breast cancer cell proliferation when the cells are cocultured with breast adipose cells with aromatase activity (Chottanapund *et al.* 2013). On the other hand, enobosarm, a nonsteroidal selective androgen receptor modulator/agonist, inhibits *in vivo* tumor growth of ERα-positive breast cancers (Ponnusamy *et al.* 2019). Consistent with these findings, immunohistochemical AR status correlates with prolonged disease-free and overall survival in ERα-positive breast cancer patients (reviewed in Venema *et al.* 2019).

Preclinical data suggest that androgens promote *in vitro* and *in vivo* tumorigenesis in ERα-negative breast cancers, especially TNBCs, while antiandrogens significantly suppress tumorigenesis (Giovannelli *et al.* 2019). These findings are consistent with increased androgen action in ERα-negative breast cancers, namely the molecular apocrine or luminal AR (LAR) subtypes (Farmer *et al.* 2005, Lehmann *et al.* 2011). In these subtypes, AR regulates a transcriptional program similar to ERα in luminal breast cancer cells. The proliferative effects of androgen on TNBC are closely associated with intracellular phosphorylation pathways, such as the MAPK pathway and the phosphatidylinositol-3 kinase (PI3K)/Akt pathway. AR activates the MAPK pathway and increases cell proliferation (Anestis *et al.* 2015). In addition, ARs form complexes with Src and PI3K and promote invasion of the TNBC cell lines, MDA-MB-231 and MDA-MB-453 (Giovannelli *et al.* 2019). Furthermore, *PIK3CA* mutations and increased Akt phosphorylation are common in TNBC, and the combined use of an AR antagonist and a PI3K inhibitor effectively suppresses the growth of TNBC cells (Lehmann *et al.* 2014). The expression of AR is posttranscriptionally regulated by PI3K, and the PI3K-mammalian target of rapamycin (mTOR) inhibitor, BEZ235, significantly decreases AR protein levels in BT549 cells (Cuenca-López *et al.* 2014). Several clinical studies targeting the AR and PI3K/Akt pathways are ongoing (Chan *et al.* 2019). Similar to ERα-positive breast cancers, AR status correlates with better clinical outcomes, but the tendency seems to be less clear than in ERα-positive cancers (Venema *et al.* 2019).

## Androgens and therapeutic resistance in breast cancer

### Resistance to SERMs

Although androgens suppress estrogen-dependent growth of ERα-positive breast cancer cells, androgens are also closely associated with resistance to endocrine therapies in breast cancers. For example, AR expression is increased in both tamoxifen-resistant cell lines and tamoxifen-resistant breast cancer tissues, and DHT promotes the proliferation of tamoxifen-resistant breast cancer cells both *in vitro* and *in vivo* (De Amicis *et al.* 2010, Chia *et al.* 2019). Furthermore, relatively higher expression of AR compared to ERα (AR:ERα ratio ≥ 2) correlates with shorter disease-free and disease-specific survival in ERα-positive breast cancers (Cochrane *et al.* 2014). On the other hand, the immunohistochemical AR status in primary breast carcinoma tissues correlates with longer disease-free and overall survival in breast cancer patients receiving endocrine therapies (SERM: 71.8%; SERM followed by AI: 25.2%; AI: 2.9%) (Park *et al.* 2012). This suggests that AR/ERα interaction, rather than pure AR action, is more closely related to resistance to tamoxifen.

### Resistance to AIs

**Figure 2:**
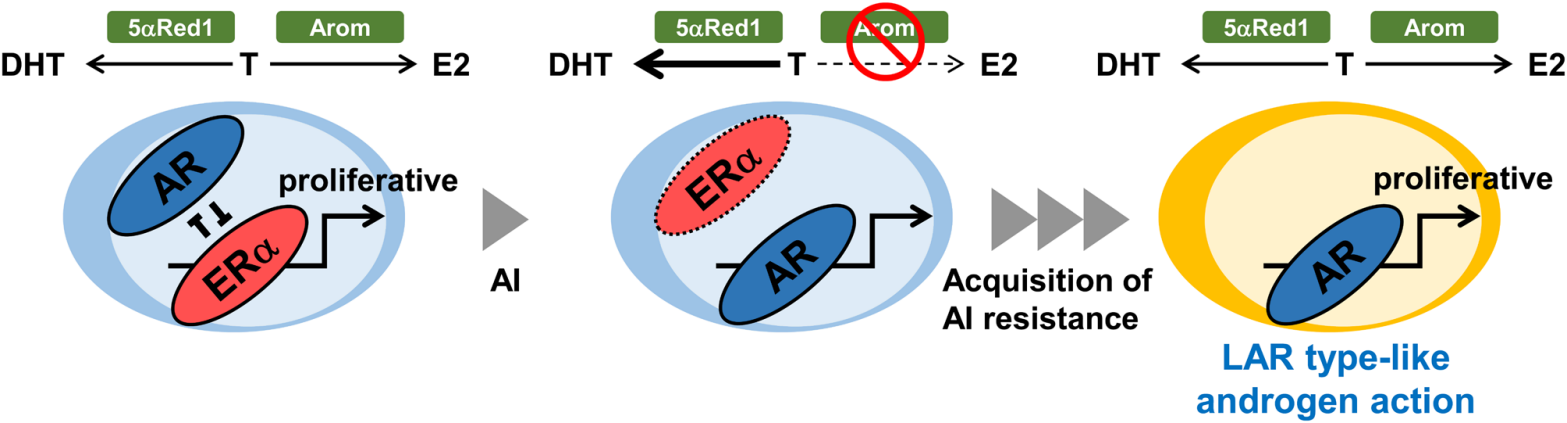
Possible relationship between androgen action and aromatase inhibitor (AI) resistance. Androgen action increases AI treatment due to increased DHT synthesis and decreased estrogen action. In the process of acquiring AI resistance, androgen action becomes similar to that of molecular apocrine or luminal androgen receptor (LAR) subtypes. Arom; aromatase.

Intratumoral DHT synthesis is increased following AI treatment, as described earlier. Therefore, several researchers investigated AR expression and its correlation with resistance to AIs. According to Chanplakorn *et al.* (2011), AR expression increases significantly after 3 months of neoadjuvant AI treatment. Furthermore, AR expression is maintained in recurrent breast cancer tissues after AI treatment, while ERα and PR expression levels are significantly downregulated (Fujii *et al.* 2014). The expression of prostate-specific antigen, a representative androgen-induced gene, is increased in recurrent tissues. In addition, a recent transcriptional analysis revealed an increased ratio of AR:ERα signaling pathway activities in patients failing AI therapy (Bleach *et al.* 2021). These findings suggest that increased androgen action is closely linked to AI resistance. However, the correlation between AR status and the efficacy of adjuvant AI treatment is still under discussion, as opposite findings have also been reported. For instance, a prospective cohort study showed that the immunohistochemical AR status in primary breast carcinoma tissues correlates with longer disease-free survival in the patients treated with AIs (Elebro *et al.* 2015). However, AR status does not serve as a predictive marker for letrozole treatment (Kensler *et al.* 2019). Considering these findings, androgen action may be increased but modified by loss of estrogen signaling in the process of acquired AI resistance. The modified androgen action may be similar to the actions in the molecular apocrine or LAR subtypes; both loss of estrogen signaling and increased androgen signaling are common in both situations (Fig. 2). Indeed, in the T-47D-derived AI-resistant model with lost ERα expression established by Fujii *et al.* (2014), the expression profile of androgen-induced genes is different from the parental T-47D cells. In addition, overexpression of AR results in resistance to AI (anastrozole) in MCF-7 cells, and AR collaborates with ERα to regulate estrogen-induced genes in resistant cells (Rechoum *et al.* 2014). On the other hand, antiandrogens (bicalutamide or enzalutamide) restore the sensitivity to AIs in an AI-resistant model (Creevey *et al.* 2019).

As described earlier, DHT is metabolized to 3β-diol by 17βHSD5. In addition, 3βHSD type 1 (3βHSD1) is also expressed in breast cancer cells and contributes to DHT metabolism to 3β-diol. Of note, 3β-diol can activate ERα activity. The expression of 3βHSD1 is increased in response to estradiol depletion, and 3β-diol promotes the proliferation of MCF-7 breast cancer cells in an ERα-dependent fashion (Hanamura *et al.* 2013). Furthermore, E10-V1 and E10-V2 cells, established by Hanamura *et al.*, are AI-resistant. The expression of 17βHSD5 and 3βHSD1 is increased, and the cells exhibit 3β-diol-dependent and ERα-dependent growth. Consistent with these findings, ERα activity correlates with 5αRed1 and 3βHSD1expression (Hanamura *et al.* 2014).

### Resistance to cyclin D/cyclin-dependent kinase (CDK) 4 and 6 inhibitors

The cyclin D/cyclin-dependent kinase (CDK) 4 and 6/retinoblastoma protein (Rb) pathway has been implicated in the proliferation of breast cancer cells. CDK4/6 inhibitors have recently become available for the treatment of metastatic ERα-positive/HER2-negative breast cancers (Pernas *et al.* 2018). However, *de novo* and acquired resistance to the drugs are common. Androgens may contribute to CDK4/6 inhibitor resistance. In a CDK4/6 inhibitor palbociclib-resistant model established by Ji *et al.* (2019), expression of AR was upregulated, while ERα signaling was suppressed. Interestingly, inhibition of AR with enzalutamide restored sensitivity to palbociclib. On the other hand, recent preclinical data demonstrated the efficacy of CDK4/6 inhibitors toward TNBCs, especially the LAR subtype. MDA-MB-453 LAR-type breast cancer cells are highly sensitive to CDK4/6 inhibitors (Asghar *et al.* 2017), and enzalutamide enhances the efficacy of palbociclib in AR-positive/RB-proficient TNBC cells (Liu *et al.* 2017).

### Resistance to radiation

Expression of AR seems to correlate with recurrence after radiation (Speers *et al.* 2017), although the *in vitro* or *in vivo* data do not support this correlation well. Interestingly, 17βHSD5, which is frequently expressed in breast carcinoma tissues and serves as a testosterone and PGF2α-producing enzyme, promotes radiation resistance in DU145 prostate cancer cells (Sun *et al.* 2016).

## Androgen action in breast cancer stromal cells

Solid tumors are composed of not only cancer cells but various types of stromal cells, including fibroblasts, lymphocytes, and macrophages. Tumor-infiltrating lymphocytes (TILs) are important in the response to chemotherapy and immune checkpoint inhibitors (Paijens *et al.* 2021), and the regulation of lymphocyte infiltration in breast carcinoma is one of the main concerns. Several studies demonstrated the correlation between AR expression and lymphocyte infiltration. For instance, AR expression is negatively correlated with CD4-positive and CD8-positive T cell infiltration in ERα-positive breast cancers (Okano *et al.* 2019). Similarly, in HER2-positive breast cancer cases, AR status is inversely correlated with CD3-positive and CD8-positive T cell infiltration. This tendency is detected in ERα-positive cases but not in ERα-negative cases (van Rooijen *et al.* 2018). Conversely, in TNBC breast cancers, a significant positive correlation between AR and CD3-positive T cell infiltration was observed (Elghazawy *et al.* 2021). These findings suggest that androgens have opposite effects on intratumoral T cell infiltration in ERα-positive and ERα-negative breast cancers. Similarly, androgens regulate TILs in prostatic hyperplasia and prostate cancers, and T cell infiltration into prostate tissues is induced by androgen deprivation therapy, suggesting negative regulatory effects of androgen on T cell infiltration in prostate tissues (Özdemir & Dotto 2019).

Intratumoral macrophages (tumor-associated macrophages; TAMs) are also a major component of the breast cancer microenvironment and promote breast cancer progression by secreting pro-tumor cytokines or chemokines (Yamaguchi *et al.* 2021*a*). A recent study showed that macrophages express AR and play pivotal roles in human diseases, including breast cancers (Yamaguchi *et al.* 2021*b*). Therefore, intratumoral DHT may affect not only breast carcinoma cells but also TAMs. Although the significance of androgen action in TAMs in breast cancers is not well understood, we previously demonstrated that the 5αRed1-positive/higher macrophage infiltration phenotype significantly correlates with worse clinical outcomes in breast cancer patients (Yamaguchi *et al.* 2021*a*). *In vitro* and *in vivo* experiments using 4T1 murine breast cancer cells and RAW264 macrophages revealed that androgens enhance the pro-tumorigenic effects of macrophages (Yamaguchi *et al.* 2021*a*). Androgen actions in the breast cancer microenvironment are important for understanding androgen actions in breast cancers.

## Membrane androgen receptors in breast cancers

**Figure 3:**
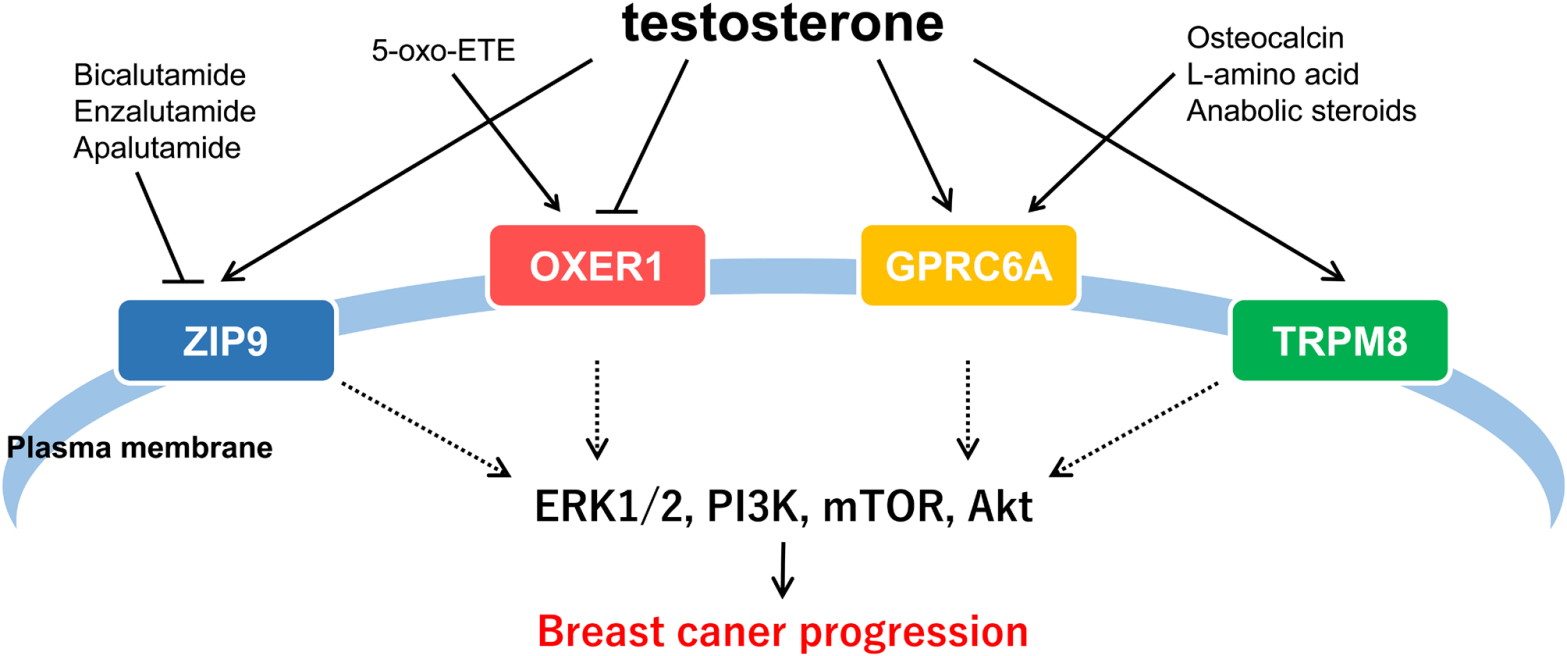
Schematic of possible roles of membrane androgen receptors. ZIP9, OXER1, GPRC6A, and TRPM8 are representative membrane androgen receptors that mediate intracellular signaling, such as MAPK (ERK1/2), PI3K, mTOR, and Akt signaling to promote breast cancer progression.

In addition to the induction of transcription by nuclear AR, androgens activate intracellular signaling by increasing second messengers such as Ca^2+^ and cAMP in a few seconds (Foradori *et al.* 2008). This nongenomic action may be mediated by cell membrane-anchored proteins (membrane-associated androgen receptors; mARs) rather than classical nuclear ARs. Activated intracellular signaling can be detected in AR-negative DU-145 and PC-3 prostate cancer cells (Thomas 2019). To date, four proteins have been identified as mARs, including ZRT-and Irt-like protein 9 (ZIP9), oxoeicosanoid receptor 1 (OXER), G protein-coupled receptor class C group 6 member A (GPRC6A), and transient receptor potential cation channel subfamily M member 8 (TRPM8) (Fig. 3).

ZIP9 is a member of the 14 ZIP protein family and serves as a transporter of Zn^2+^ across membranes from the extracellular compartment into the cytoplasm or from the cytoplasm into organelles, such as the Golgi body (Cousins *et al.* 2006). ZIP9 is a mAR in the ovary of Atlantic croaker (Braun & Thomas 2003), and testosterone has a higher affinity for ZIP9 than DHT or androstenedione (Thomas *et al.* 2018). ZIP9 is coupled to inhibitory Gα proteins (Gαi) and activates the MAPK (extracellular signal-regulated kinase (ERK) 1/2) pathway to promote tumors (Aguirre-Portolés *et al.* 2021). Interestingly, this tumorigenic effect is inhibited by classic antiandrogens, such as bicalutamide, enzalutamide, and apalutamide (Aguirre-Portolés *et al.* 2021). ZIP9 is expressed at a higher level in breast cancer cell lines than other membrane androgen receptors (Kalyvianaki *et al.* 2019). Although the role of ZIP9 in breast cancers is unclear, ZIP9 is overexpressed in breast carcinoma tissues and predicts worse prognoses in HER2-type breast cancers (Liu *et al.* 2020).

OXER1 is a G protein-coupled receptor activated by 5-oxoeicosatretraenoic acid (5-oxo-ETE), which is produced by 5-lipoxygenase, resulting in increased breast cancer cell proliferation (Avis *et al.* 2001). OXER1 promotes the migration of several cancer cell lines, including the T-47D breast cancer cell line (Kalyvianaki *et al.* 2021). Although the expression of OXER1 has not been thoroughly examined in breast cancer tissues, recent RNA-sequence analysis demonstrated that OXER1 expression is higher in ERα-negative breast cancers than in ERα-positive breast cancers (Masi *et al.* 2020). OXER1 may also serve as a molecular marker in ERα-positive/HER2-negative breast cancers of adolescents and young adults (Yi & Zhou 2020). On the other hand, testosterone acts as an antagonist of OXER1-mediated intracellular signaling (Kalyvianaki *et al.* 2017). Thus, androgens antagonize the effects of 5-oxo-ETE on OXER1.

GPRC6A is a G protein-coupled receptor activated by multiple ligands, including osteocalcin, l-amino acid, and anabolic steroids (Pi & Quarles 2012). Therefore, GPRC6A acts as a sensor of anabolic responses in multiple tissues. GPRC6A mediates the nongenomic actions of steroids, such as testosterone, and testosterone promotes ERK phosphorylation in HEK293 cells that stably express GPRC6A but not in control HEK293 cells (Pi *et al.* 2010). Of note, this activation was observed for both free testosterone and BSA-bound testosterone, which cannot penetrate the cell. The tumorigenic effects of GPRC6A have been widely studied in prostate cancer. GPRC6A expression levels are higher in VCaP and PC3 prostate cancer cells compared with expression levels in RWPE-1 normal prostate cells (Liu *et al.* 2016). In addition, GPRC6A promotes prostate cancer cell proliferation and migration by activating ERK and mTOR phosphorylation in response to testosterone and osteocalcin (Ye *et al.* 2017). To the best of our knowledge, the role of GPRC6A in breast cancer has not been examined. However, L-arginine promotes mammary epithelial cell proliferation and development of mammary glands in pubertal mice through GPRC6A/PI3K/AKT/mTOR signaling (Ge *et al.* 2022). In humans, *GPRC6A* gene polymorphisms have been reported, consisting mostly of KGKY insertion/deletion in the third intracellular loop (GPRC6A^ICL3_KGKY^) (Ye *et al.* 2019). These polymorphisms are gain-of-function polymorphisms (Pi *et al.* 2020).

Activation of TRPM8 ion channels causes Na^+^ and Ca^2+^ entry into the cells (Andersen *et al.* 2014). TRPM8 is activated at 15–25°C and functions as a transducer of cold temperature (Yee 2015). TRPM8 interacts with testosterone and testosterone activates a TRPM8-mediated Ca^2+^ response (Asuthkar *et al.* 2015). Interestingly, classic AR interacts with TRPM8 and modulates channel activity, suggesting the interplay between the membrane and classic ARs (Grolez *et al.* 2019). TRPM8 promotes EMT in breast cancer cells (Liu *et al.* 2014), and N-(3-aminopropyl)-2-{[(3-methylphenyl) methyl]oxy}(20)-N-(2-thienylmethyl)benzamide, a TRPM8 inhibitor, suppresses proliferation and migration of breast cancer cell lines (Yapa *et al.* 2018). In addition, expression of TRPM8 may be increased and is associated with larger tumor size (Liu *et al.* 2014), especially in breast carcinoma tissues, and TRPM8 is expressed more often in basal type breast cancers compared with the expression in other subtypes (Yapa *et al.* 2018).

## Conclusion

The actions of androgen in breast cancers are still largely unclear, although they have been the focus of many investigations. However, a growing body of evidence supports the association of androgen with breast cancer biology. The integration of knowledge from past and recent studies is important for improving endocrine therapies and identifying new therapeutic targets in androgen-dependent breast cancers.

## Declaration of interest

The authors declare that there is no conflict of interest that could be perceived as prejudicing the impartiality of this review.

## Funding

This work was partly supported by JSPS KAKENHI Grant number 19K09065 and 19K07410.
